# Impact of EV administration on murine hematology and metabolism

**DOI:** 10.7717/peerj.20952

**Published:** 2026-03-23

**Authors:** Hongyu Qin, Suliang Li, Tianyu Wang, Xi Wang, Yuhan Sun, Pengxiang Qu, Shengyu Wang, Yongli Zhou, Yun Ye

**Affiliations:** 1 Central Laboratory, The First Affiliated Hospital of Xi’an Medical University, Xi’an Medical University, Xi’an, China; 2 Department of Blood Transfusion, the First Affiliated Hospital of Xi’an Medical University, Xi’an Medical University, Xi’an, China; 3 Laboratory Animal Center, Xi’an Jiaotong University Health Science Centre, Xi’an Jiaotong University, Xi’an, China; 4 Department of Respiratory and Critical Care Medicine, the First Affiliated Hospital of Xi’an Medical University, Xi’an Medical University, Xi’an, China; 5 Department of Gastroenterology, the First Affiliated Hospital of Xi’an Medical University, Xi’an Medical University, Xi’an, China

**Keywords:** Extracellular vesicles, Xenotransplantation, Syngeneic transplantation, Metabolism, Immunogenicity

## Abstract

Extracellular vesicles (EVs), promising natural nanocarriers, face clinical hurdles like heterogeneity and immunogenicity. This study evaluates the species-specific effects and metabolic impacts of EV administration, aiming to explore their therapeutic value. EVs were isolated from rabbit and C57BL/6 mouse *via* ultracentrifugation, with characterization performed using transmission electron microscopy and nanoparticle tracking analysis. EVs were administered *via* tail vein injection to C57BL/6 mice and ob/ob mice, followed by longitudinal monitoring of blood biochemical parameters, hematological profiles, and hepatic pathological alterations. Quantitative polymerase chain reaction (qPCR) was employed to analyze EV-associated miRNA expression and associated target gene regulatory mechanisms. The study revealed that syngeneic EVs induced transient physiological fluctuations during acute exposure, with no significant alterations in blood parameters after chronic intervention. Xenogeneic EVs triggered elevated alkaline phosphatase and leukocyte imbalance, suggesting immune activation. Healthy donor EVs ameliorated hepatic steatosis in ob/ob mice, coinciding with enriched miR-26 levels in donor EVs and recipient livers. This study shows the safety benefits of syngeneic EVs, while noting the immunogenic risks of xenogeneic EV administration. Healthy donor EVs significantly reduced hepatic steatosis in obese mice, supporting the clinical translation of EV therapies.

## Introduction

Extracellular vesicles (EVs), functioning as natural nanocarriers, show promise in disease treatment and regenerative medicine due to their low immunogenicity, ability to traverse biological barriers, and capacity to transport bioactive compounds (Kumar et al., 2024). In recent years, EVs have been explored as drug delivery systems (Aslan et al., 2021), immunomodulators, and inducers of tissue healing (Luo et al., 2021). Their bioactive components (miRNAs and proteins) may influence disease processes by modulating signaling pathways in target organs. However, clinical translation faces challenges due to functional variability and unresolved immunogenicity risks in xenogeneic applications (Younis et al., 2022). Recent studies suggest syngeneic EVs, owing to species-conserved surface proteins and nucleic acids, may exhibit improved biocompatibility (Rayamajhi & Aryal, 2020; Kim et al., 2024); yet their long-term safety and metabolic regulatory mechanisms require further validation.

Although preclinical studies indicate EV-based therapeutics alleviate tissue damage and aging (Tian et al., 2023; You et al., 2023), clinical implementation encounters challenges. Current research largely focuses on *in vitro* studies and delivery efficacy, while data on short-term effects or long-term immunological/organ accumulation risks remain limited (Gupta, Zickler & El Andaloussi, 2021). Mesenchymal stem cell-derived EVs have been reported to mitigate diabetic nephropathy; however, repeated administration may pose risks of unspecified immunotoxicity (Zheng, Wang & Wang, 2024; Sun et al., 2022; Li et al., 2024). These observations highlight technical and mechanistic hurdles in translating EV therapies from bench to bedside.

In metabolic diseases, EV-derived miRNAs are increasingly regarded as important regulators (Zhao et al., 2021; Negi, Rutman & Paraskevas, 2019). Conditions like obesity and non-alcoholic fatty liver disease (NAFLD) have been associated with altered circulating EV miRNA profiles (Ji & Guo, 2019; Hochreuter et al., 2022). These miRNAs may modulate lipid synthesis, insulin sensitivity, and inflammation by targeting pathways in liver and adipose tissues. For instance, miR-122 and miR-34a appear involved in hepatic steatosis regulation, while the miR-26 family has been observed to improve metabolic imbalance through suppression of lipogenic genes (*e.g.*, SREBP1). Nevertheless, animal studies exploring EV administration for metabolic improvement are limited, constraining comprehensive assessment of their therapeutic potential.

## Materials & Methods

### Experimental animals

The experimental animals used in this study included New Zealand White rabbits, C57BL/6 mice, and ob/ob mice (C57BL/6 background). All rabbit and mouse experiments were approved by the Animal Ethics Committee of Xi’an Jiaotong University(XJTULAC201936751) and conducted in accordance with institutional guidelines. Two-month-old male rabbits and 7-week-old male C57BL/6J mice were obtained from the Laboratory Animal Centerr of Xi’an Jiaotong University. Animals were housed under controlled temperature conditions (22–24 °C) with a 12-hour light/dark cycle and provided ad libitum access to food and water (Andre et al., 2018). Mice were group-housed (≤5/cage) and rabbits individually housed in standard cages with aspen chip bedding, nesting material, and tunnels for enrichment. All surgical and terminal procedures were performed under anesthesia. Mice undergoing terminal procedures were deeply anesthetized with isoflurane (5% induction, 2% maintenance). Retro-orbital bleeding was conducted under brief isoflurane anesthesia. Tail vein injection, deemed minimally invasive, did not require analgesia. Any animal showing severe distress would have been euthanized immediately, though none met these criteria before the planned endpoint. All animals were euthanized at the study’s conclusion: mice by cervical dislocation under deep isoflurane anesthesia, and rabbits by intravenous overdose of sodium pentobarbital (150 mg/kg). Death was confirmed by absent respiration, cardiac activity, and corneal reflex.

### Isolation and characterization of EVs

All animals were fasted for 12 h prior to the experiment. Plasma samples were collected from rabbits and C57BL/6 mice *via* cardiac puncture using 21-gauge needles (rabbits) or retro-orbital bleeding using heparinized capillaries (mice). All samples were immediately mixed with 0.38% sodium citrate (1:9 v/v) and centrifuged at 2,000 × g for 15 min at 4 °C to obtain platelet-poor plasma, followed by storage at −80 °C. EVs were isolated following the standardized protocol recommended by the International Society for Extracellular Vesicles (ISEV), utilizing ultracentrifugation as the primary isolation method. First, plasma samples were centrifuged at 3,000 × g for 15 min at 4 °C to remove cellular debris. The supernatant was then centrifuged at 10,000 × g for 30 min at 4 °C to eliminate large particles. Finally, the supernatant was ultracentrifuged at 100,000 × g for 70 min at 4 °C to pellet EVs. The pellet was resuspended in PBS and ultracentrifuged again at 100,000 × g for 70 min to remove contaminants (Wu et al., 2019; Qu et al., 2022). For morphological and size analysis, the EV suspension was dropped onto a copper grid, negatively stained with 2% phosphotungstic acid, and observed under a transmission electron microscope (Wu et al., 2019; Qu et al., 2022; Pascucci & Scattini, 2021). The particle size distribution and concentration of the EV suspension were analyzed using a Nanosight NS300 instrument. The EV dose (2.0 × 10^10^ particles/mL) for administration was selected based on concentrations commonly reported to elicit biological effects in prior *in vivo* studies of EV therapeutics (Gupta, Zickler & El Andaloussi, 2021; Xu et al., 2023; Driedonks et al., 2022). We acknowledge that the UC isolation method may co-isolate non-vesicular contaminants, such as protein aggregates. The washing step with PBS was included to mitigate this limitation.

### Administration of EVs

Prior to EV administration, ultracentrifugation-purified EVs (derived from rabbits or mice) were diluted in sterile PBS to a target concentration (2.0 × 10^10^ particles/mL), vortex-mixed, and temporarily stored on ice. Experimental mice (C57BL/6 or ob/ob) were placed in mouse restrainers, and their tails were disinfected with 75% ethanol. A 30-gauge insulin injection needle (BD Biosciences) was used to gently puncture the lateral tail vein, followed by slow injection of 100 µL EV suspension. Post-injection, pressure was applied with a cotton ball to achieve hemostasis (Liu et al., 2022; Morales-Prieto et al., 2022).

### Hematological and biochemical blood analysis

Whole blood samples were analyzed using a Sysmex XN-1000 analyzer to determine white blood cell (WBC), red blood cell (RBC), hemoglobin (HGB), platelet (PLT) counts, and lymphocyte (LY%)/neutrophil (NE%) ratios (Patel et al., 2025). Serum samples were analyzed *via* a Hitachi 7180 automated biochemistry analyzer to measure alanine aminotransferase (ALT), aspartate aminotransferase (AST), alkaline phosphatase (ALP), urea nitrogen (BUN), creatinine (CRE), triglycerides (TG), and total cholesterol (TC) (Fernandes et al., 2018).

### Histopathological analysis

All murine histological procedures were performed under a double-blinded design, with experimental technicians fully blinded to group assignments. Tissue samples were fixed in 4% neutral-buffered formalin, dehydrated through an ethanol gradient (70%, 80%, 95%, 100%), and cleared in xylene. Paraffin infiltration was completed using a fully automated VIP5/VIP6 tissue processor (TissueTek; Sakura-Americas) (Hansen et al., 2020). After embedding with a Histostar automated embedding workstation (Thermo Fisher Scientific), four µm-thick consecutive sections were prepared using an M355S rotary microtome (Thermo Fisher Scientific) and mounted on glass slides. Hematoxylin and eosin (H&E, Thermo Fisher Scientific) staining was performed, followed by pathological evaluation strictly adhering to the NAFLD Activity Score (NAS) criteria (Qu et al., 2023).

### Hepatic lipid content quantification

Hepatic lipid extraction was performed using the chloroform-methanol method coupled with enzymatic biochemical quantification. Fresh or −80 °C-stored liver tissue (approximately 50 mg) was homogenized in ice-cold PBS (pH 7.4) at a 1:9 (w/v) tissue-to-buffer ratio. A 200 µL aliquot of the homogenate was mixed with chloroform-methanol (2:1, v/v; total volume four mL), vortexed for 5 min, and incubated at 4 °C for 12 h to facilitate phase separation. After centrifugation (3,000 ×g, 15 min, 4 °C), the lower organic phase was collected, dried under nitrogen gas, and reconstituted in 1% Triton X-100/PBS solution. Total triglycerides (TG) concentration was quantified using assay kits (Cat# A110-1-1 and A111-1-1; Nanjing Jiancheng Bioengineering Institute) according to manufacturer protocols. Reaction mixtures were prepared as specified, and absorbance values were measured using a microplate reader (BioTek Synergy H1). Lipid concentrations were calculated against standard curves and normalized to tissue weight (µg/mg tissue).

### Quantitative polymerase chain reaction

The extraction of EV miRNA was performed using Trizol LS reagent (Invitrogen) in combination with the miRNeasy Mini Kit (Qiagen). Purified EV particles were lysed with one mL Trizol LS, followed by phase separation with chloroform (centrifugation at 12,000 ×g for 15 min at 4 °C). The aqueous phase was collected and mixed with 1.25 volumes of absolute ethanol, followed by purification through the miRNeasy silica membrane column. Residual genomic DNA was removed using DNase I (Qiagen), and RNA was eluted in 30 µL of RNase-free water. RNA quality was assessed using a Nanodrop 2000 spectrophotometer (Thermo Fisher Scientific) to determine purity (A260/A280 > 1.8), and integrity was verified using an Agilent 2100 Bioanalyzer (RIN value ≥ 7.0). Reverse transcription was performed using the TaqMan MicroRNA Reverse Transcription Kit (Applied Biosystems). Total RNA was mixed with miRNA-26a-specific stem-loop primers and dNTPs, denatured at 70 °C for 5 min, and then placed on ice to terminate the reaction. MultiScribe™ Reverse Transcriptase and RNase inhibitor were added, and the mixture was incubated at 42 °C for 60 min, followed by inactivation at 85 °C for 5 min to generate cDNA.

For miRNA extraction from liver tissue, the TRIzol method was employed. RNA concentration and A260/A280 ratio were measured using a UV spectrophotometer. Poly(A)-tailing total RNA, followed by RT with an oligo-dT adapter primer. Quantitative polymerase chain reaction (qPCR) uses a miRNA-specific forward primer and a universal reverse primer with SYBR Green detection. For mRNA extraction from liver tissue, the TRIzol method was also used. Extracted total RNA was treated with DNase I to remove genomic DNA. One microgram of RNA was reverse transcribed using the Evo M-MLV RT mix with gDNA-Clean for qPCR Kit (Cat. No. AG11728, Accurate Biology). RNA was incubated at 65 °C for 5 min to disrupt secondary structures, followed by cDNA synthesis at 42 °C for 15 min. qPCR was performed using the SYBR^®^ Green Premix Pro Taq HS Kit (Cat. No. AG11701; Accurate Biology). A 20 µL reaction system containing target gene-specific primers was prepared, and Ct values were determined using a real-time fluorescence quantitative polymerase chain reaction (PCR) instrument. The expression levels of miRNA or mRNA were calculated based on the obtained Ct values (Bustin et al., 2009).

### Statistical analysis

Randomized group assignment with blinded assessment. Data are presented as mean ± standard error of the mean. Statistical analyses were performed using GraphPad Prism 9.0. One-way analysis of variance (ANOVA) followed by Tukey’s multiple comparison test was applied for comparisons among multiple groups, while unpaired t-tests were used for comparisons between two groups. Statistical significance was defined as *p < 0.05, **p < 0.01, ***p < 0.001, and ****p < 0.0001. Heatmaps were performed using the OmicStudio tools, Z-score normalization was applied to each biochemical parameter (column).

## Results

### Morphological characteristics of plasma-derived EVs from rabbits, C57BL/6, and ob/ob Mice

**Figure 1:**
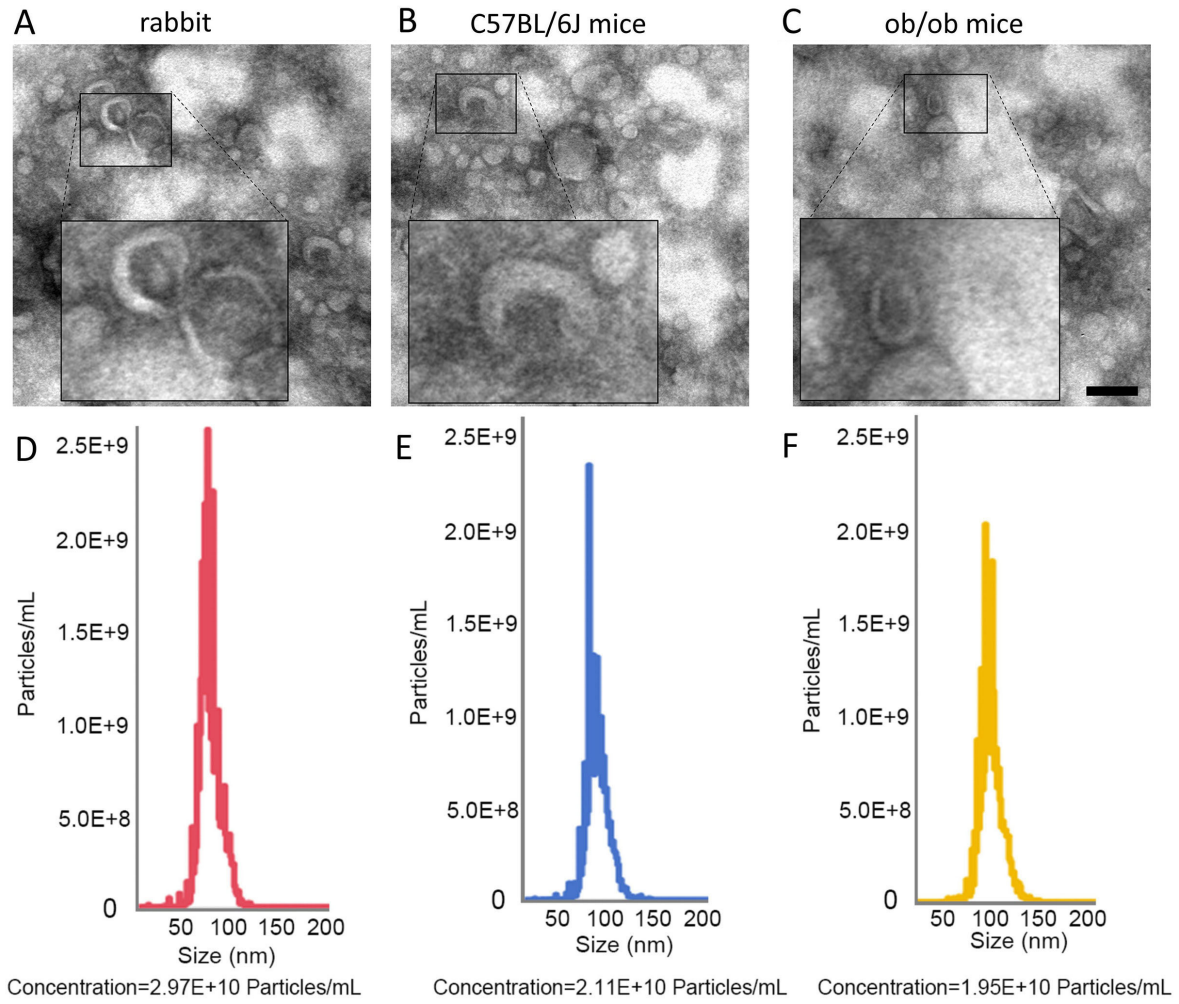
Morphological characterization of plasma EVs from rabbits, C57BL/6 mice, and ob/ob mice. (A–C) Transmission electron microscopy (TEM) images of plasma EVs from rabbits (A), C57BL/6J mice (B), and ob/ob mice (C). Scale bar = 100 nm. (D–F) Nanoparticle tracking analysis (NTA) of plasma EVs from rabbits (D), C57BL/6J mice (E), and ob/ob mice (F), showing size distribution and particle concentration. *X*-axis: particle size (nm); *Y*-axis: particle concentration (particles/mL).

The morphological features and concentrations of plasma-derived EVs from different sources were systematically characterized using transmission electron microscopy (TEM) and nanoparticle tracking analysis. TEM observations revealed that EVs derived from rabbit, C57BL/6, and ob/ob mice (C57BL/6 background) exhibited a round or oval shape with a typical cup-like morphology (Figs. 1A–1C). Nanoparticle tracking analysis indicated that the particle size of EVs from all three sources primarily ranged between 50–150 nm, consistent with the standard size range for EVs. Rabbit plasma EVs exhibited a concentration of 2.97 × 10^10^ particles/mL, followed by C57BL/6 mice with 2.11 × 10^10^ particles/mL, while ob/ob mice showed a concentration of 1.95 × 10^10^ particles/mL (Figs. 1D–1F).

### Blood parameter abnormalities and inflammatory responses induced by heterologous EV administration

**Figure 2:**
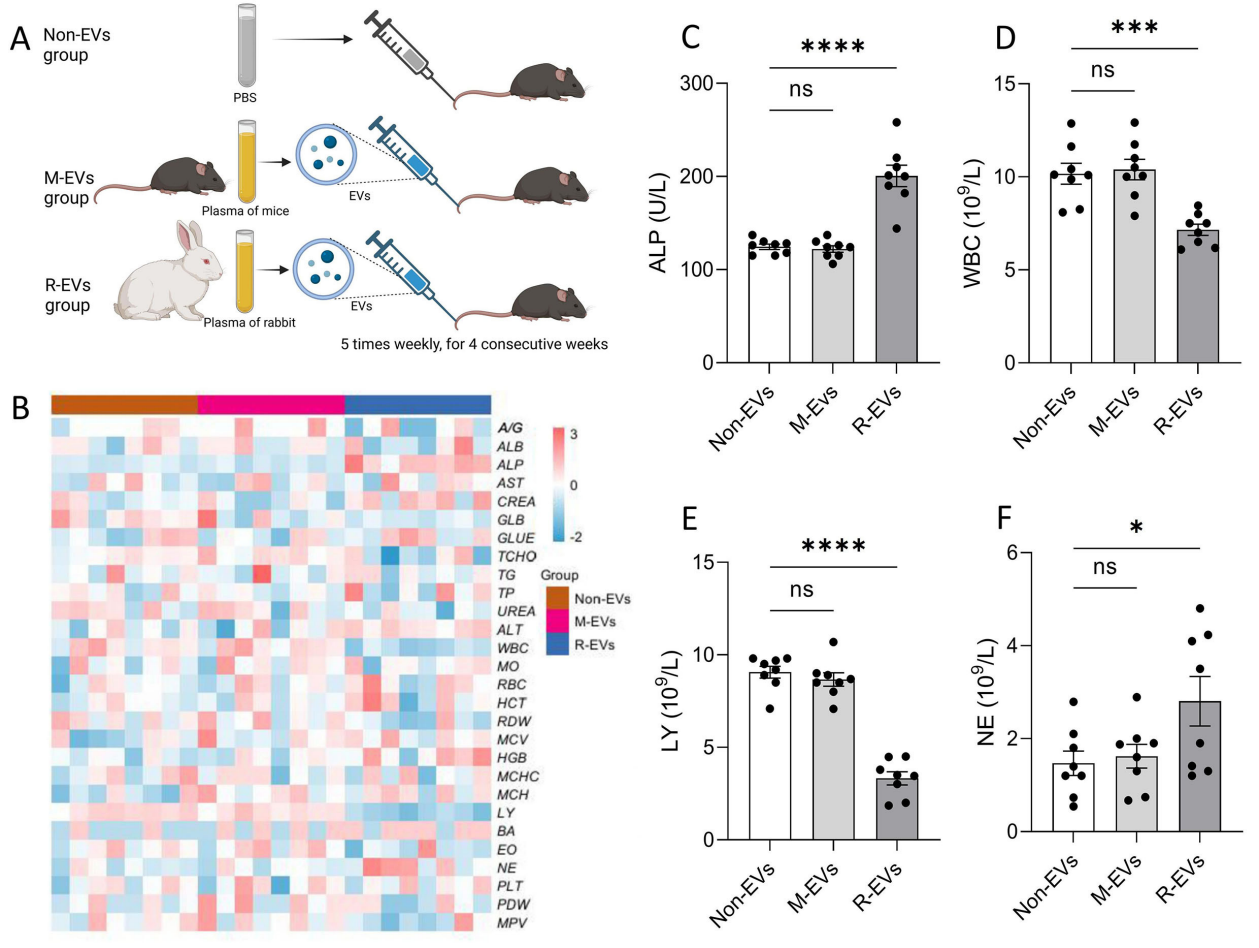
Xenogeneic exosome administration-induced hematological abnormalities and inflammatory responses. (A) Experimental design: C57BL/6 mice were randomly allocated into three groups (*n* = 8/group) receiving consecutive 4-week intraperitoneal injections (five times/week) of: (1) M-EVs (syngeneic administration): 100 u L C57BL/6-derived EVs (2.0×10 10 particles/mL); (2) R-EVs (xenogeneic administration): 100 u L New Zealand rabbit-derived EVs (2.0×10 10 particles/mL); (3) Non-EVs (PBS as control): 100 u L PBS. (B) Heatmap of blood biochemical and hematological parameters across groups. Color intensity reflects relative levels of measured parameters, including A/G, ALB, ALP, AST, CREA, GLB, GLUO, TCHO, TG, TP, UREA, ALT, WBC, MO, RBC, HCT, RDW, MCV, HGB, MCHC, MCH, LY, BA, EO, NE, PLT, PDW, MPV. (C–F) Significantly altered parameters: ALP (C), WBC (D), LY (E), and NE (F) levels. ****p < 0.0001; ***p < 0.001; *p < 0.05 *vs.* control.

To evaluate the potential biological effects of cross-species EV administration, the impact of EVs from different sources on recipient mice was analyzed through long-term injection experiments. C57BL/6 mice were randomly divided into three groups (*n* = 8 per group) and tail vein injections (100 µL, 5 times per week for 4 weeks): (1) M-EVs group (homologous administration): injected with C57BL/6-derived EVs (2.0 × 10^10^ particles/mL); (2) R-EVs group (heterologous administration): injected with New Zealand rabbit-derived EVs (2.0 × 10^10^ particles/mL); (3) non-EVs group (PBS as control): injected with an equal volume of PBS (Fig. 2A). Post-intervention, systemic effects of EVs were assessed through blood biochemistry and hematology. Results showed no significant differences between the Non-EVs and M-EVs groups in all measured parameters, including liver function enzymes (ALT, AST), renal function markers (BUN, CRE), lipid metabolism markers (TG, TC), and hematological parameters (WBC, RBC, HGB, PLT) (Fig. 2B), indicating that long-term homologous EV administration did not induce detectable toxicity or interference in the recipient mice’s blood system or metabolic functions.

Compared to the M-EVs group, the R-EVs group showed no significant differences in most parameters (*e.g.*, ALT, AST, BUN, CRE, TG, TC, RBC, HGB, PLT). However, ALP levels were significantly elevated, suggesting hepatobiliary injury and liver dysfunction. Additionally, total WBC counts significantly decreased, lymphocyte percentages (LY%) significantly declined, and neutrophil percentages (NE%) significantly increased (Figs. 2C–2F), indicating that heterologous EV administration may induce inflammatory responses. The result reveal that long-term homologous EV administration (C57BL/6 to C57BL/6) exhibits high compatibility, while heterologous EV administration (rabbit to mouse) specifically alters certain blood parameters (*e.g.*, ALP, WBC subsets), highlighting immunogenicity risks that may be compounded by co-isolated contaminants.

### No significant changes in blood physiology following homologous EV administration

**Figure 3:**
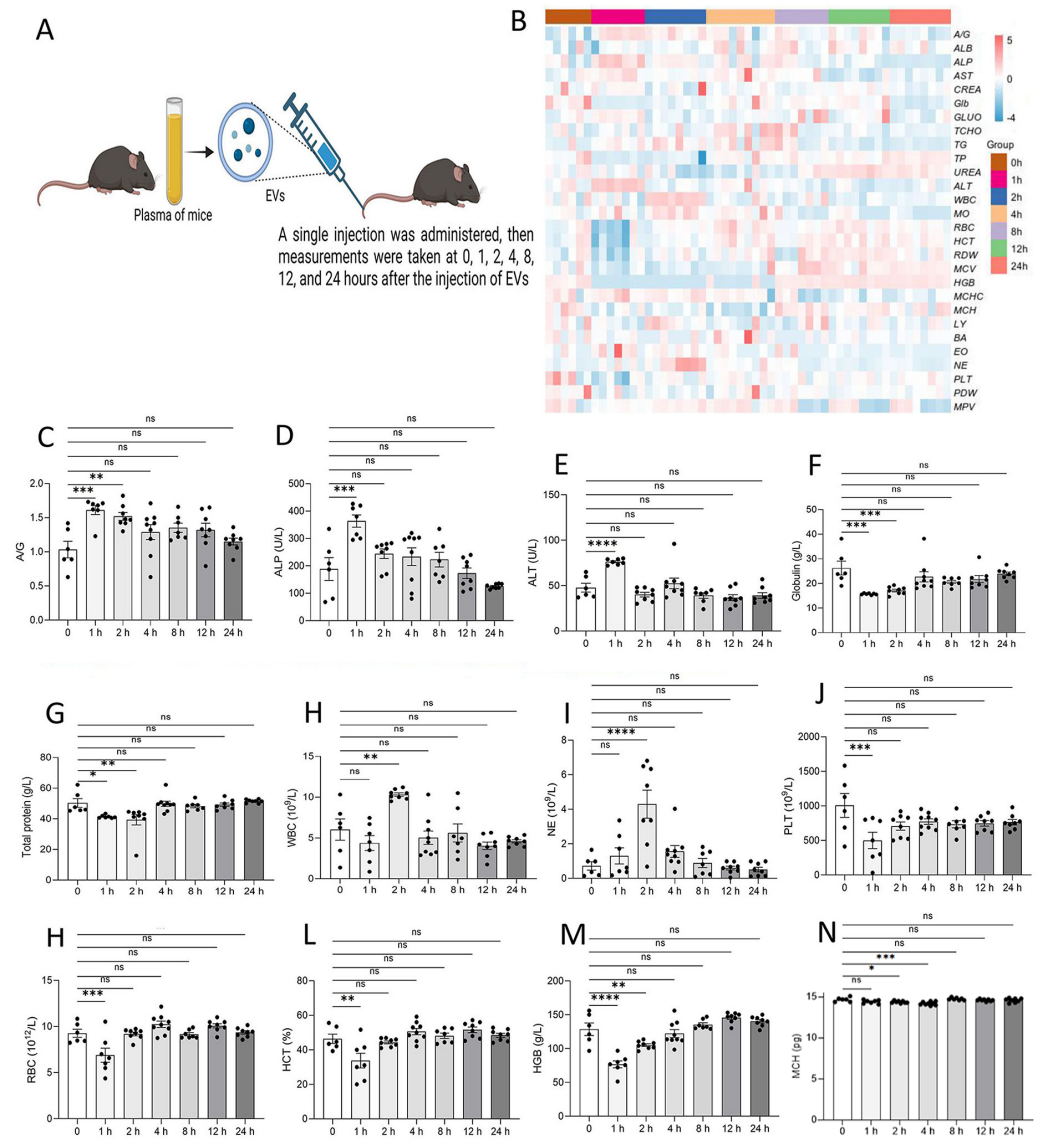
Short-term effects of syngeneic exosome administration in mice. (A) Experimental design: C57BL/6 mice received a single tail vein injection of 100 u L syngeneic EVs (2.0×10 10 particles/mL). Blood parameters were dynamically monitored at 0, 1, 2, 4, 8, 12, and 24 h post-injection (*n* = 6–8 in each point, with separate groups per time point). (B) Heatmap of temporal changes in blood biochemical and hematological parameters. (C–N) Significantly altered parameters: A/G (C), ALP (D), ALT (E), Glu (F), total protein (G), WBC (H), NE (I), PRT (J), RBC (K), HCT (L), HbFe (M), MCH (N). ns, not significant; ***p < 0.001; ****p < 0.0001; *p < 0.05 *vs.* pre-injection control.

To investigate the immediate effects of acute EV exposure, C57BL/6 mice were subjected to a single tail vein injection of 50 µL homologous EVs (2.0 × 10^10^ particles/mL), and blood biochemical parameters were dynamically monitored at 0, 1, 2, 4, 8, 12, and 24 h post-injection (Fig. 3A). Results showed no significant differences in most parameters, including liver function enzymes (ALT, AST,ALP,A/G, ALB, TCHO), renal function markers (CREA, UREA), and lipid metabolism markers (TG, TCHO), within 24 h (Fig. 3B). However, transient fluctuations in certain parameters (*e.g.*, ALP, WBC subsets) were observed at 1-4 h post-injection (Figs. 3C–3N), though all fluctuations remained within physiological reference ranges. All altered parameters returned to baseline levels within 8-24 h, confirming that short-term EV exposure did not induce persistent metabolic disturbances or blood biochemical abnormalities. These results suggest that homologous EV administration in the acute phase only induces transient and reversible physiological fluctuations, indicating good short-term biocompatibility and rapid metabolic homeostasis restoration.

**Figure 4:**
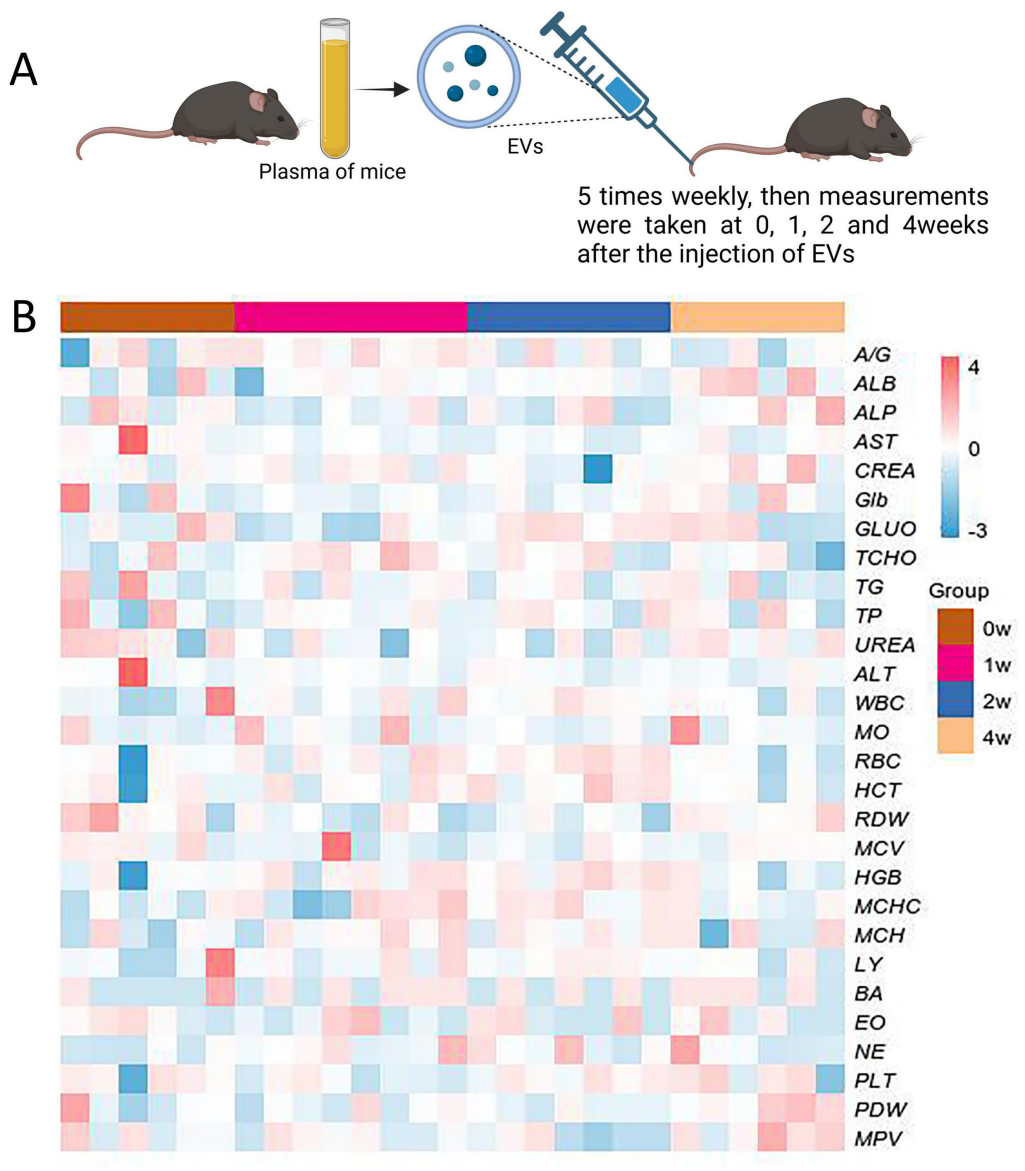
Long-term effects of syngeneic exosome administration in mice. (A) Experimental design: C57BL/6 mice underwent consecutive 4-week tail vein injections (five times/week) of 100 u L syngeneic EVs (2.0×10 10 particles/mL). Blood parameters were analyzed at 1, 2, and 4 weeks post-treatment (*n* = 6–8 in each point, with separate groups per time point). (B) Heatmap of blood biochemical and hematological parameters across timepoints.

To further assess the long-term cumulative effects of EVs, C57BL/6 mice were subjected to continuous tail vein injections of homologous EVs (50 µL per injection, 2.0 × 10^10^ particles/mL) five times per week for 4 weeks (Fig. 4A). Blood biochemical parameters were systematically analyzed at 1, 2, and 4 weeks post-intervention. Results showed no significant differences in all measured parameters between the EVs-treated group and the control group at all-time points (Fig. 4B). EV injections administered over a 4-week period did not induce any significant trend changes or progressive deviations in the measured parameters, highlighting a high tolerance to long-term homologous EV exposure.

In summary, short-term dynamic monitoring and long-term cumulative effect analyses indicate that homologous EV administration only induces transient and reversible physiological fluctuations in the acute phase. These findings further support the safety advantages of homologous EVs in therapeutic delivery, providing critical experimental evidence for their clinical translation.

### Healthy donor-derived EV administration improves hepatic metabolic function in obese model mice

**Figure 5:**
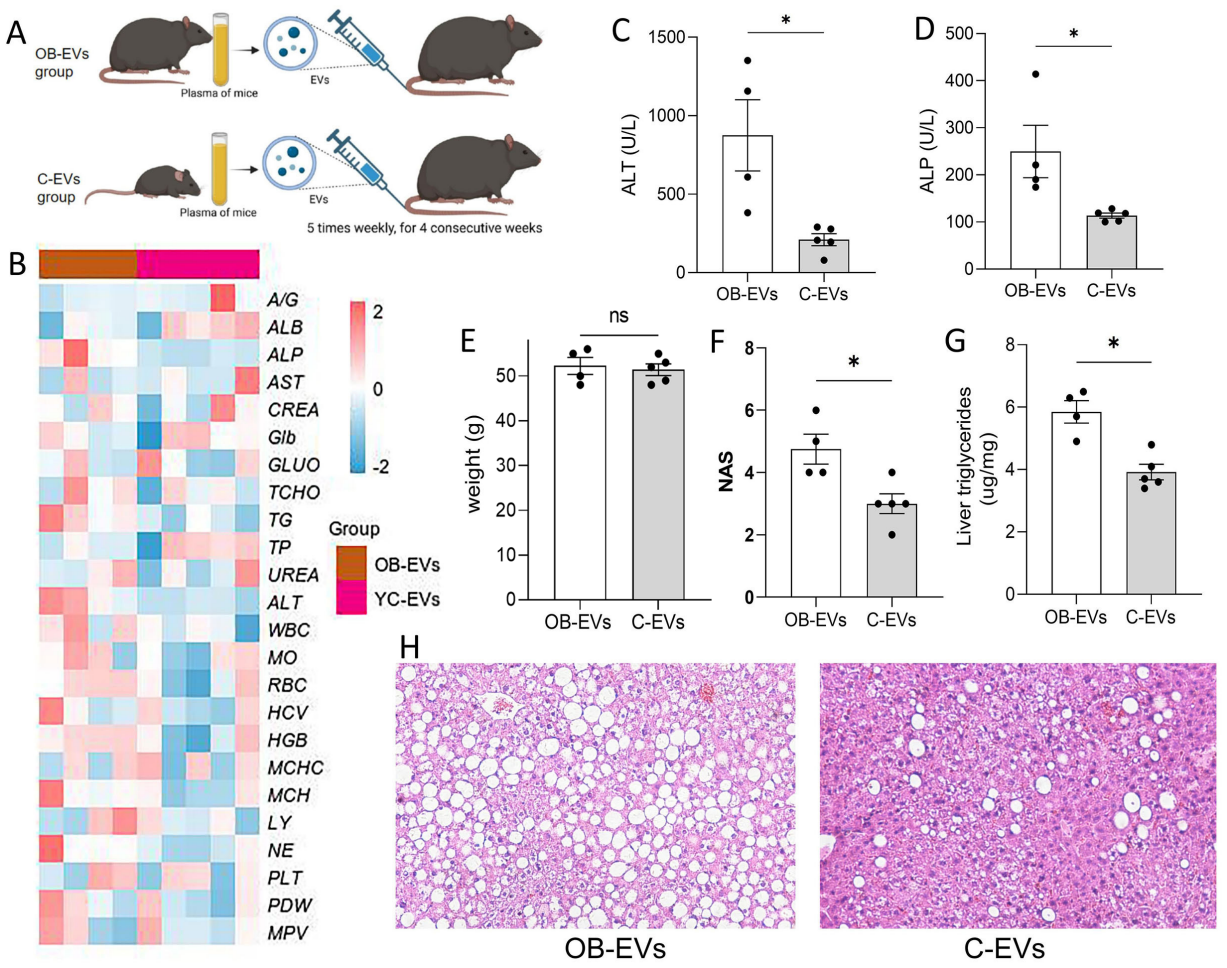
Healthy EVs administration ameliorates hepatic metabolic dysfunction in obese mice. (A) Experimental design: ob/ob mice were randomized into OB-EVs (100 u L ob/ob-derived EVs) and C-EVs (100 u L wild-type C57BL/6-derived EVs) groups, *n* = 4–5/per group, receiving 4-week tail vein injections (five times/week). (B) Heatmap of blood parameter profiles between groups. (C–D) Serum ALT (C) and ALP (D) levels. (E–G) Terminal body weight (E), MASH histopathology scores (F), and hepatic lipid content (G). (H) Representative liver histopathological images.

To explore the therapeutic potential of EVs in metabolic diseases, obese ob/ob mice were randomly divided into two groups: the OB-EVs group (receiving EVs derived from ob/ob mice, serving as disease-state EVs, 2.0 × 10^10^ particles/mL) and the C-EVs group (receiving EVs derived from wild-type C57BL/6 mice, representing healthy donor EVs, 2.0 × 10^10^ particles/mL). Both groups were subjected to continuous injections (five times per week for 4 weeks), and metabolic phenotypes and liver function were evaluated (Fig. 5A). Blood biochemical analysis revealed that serum ALT and ALP levels in the C-EVs group were significantly lower than those in the OB-EVs group (Figs. 5B–5D). Although no significant difference in body weight was observed between the two groups, the NAS score in the C-EVs group was significantly lower than that in the OB-EVs group, and the hepatic lipid accumulation area was significantly reduced, demonstrating that healthy EVs improved hepatic lipid metabolism disorders and steatosis (Figs. 5E–5I). This finding provides experimental evidence for intervention strategies based on EVs in metabolic diseases.

**Figure 6:**
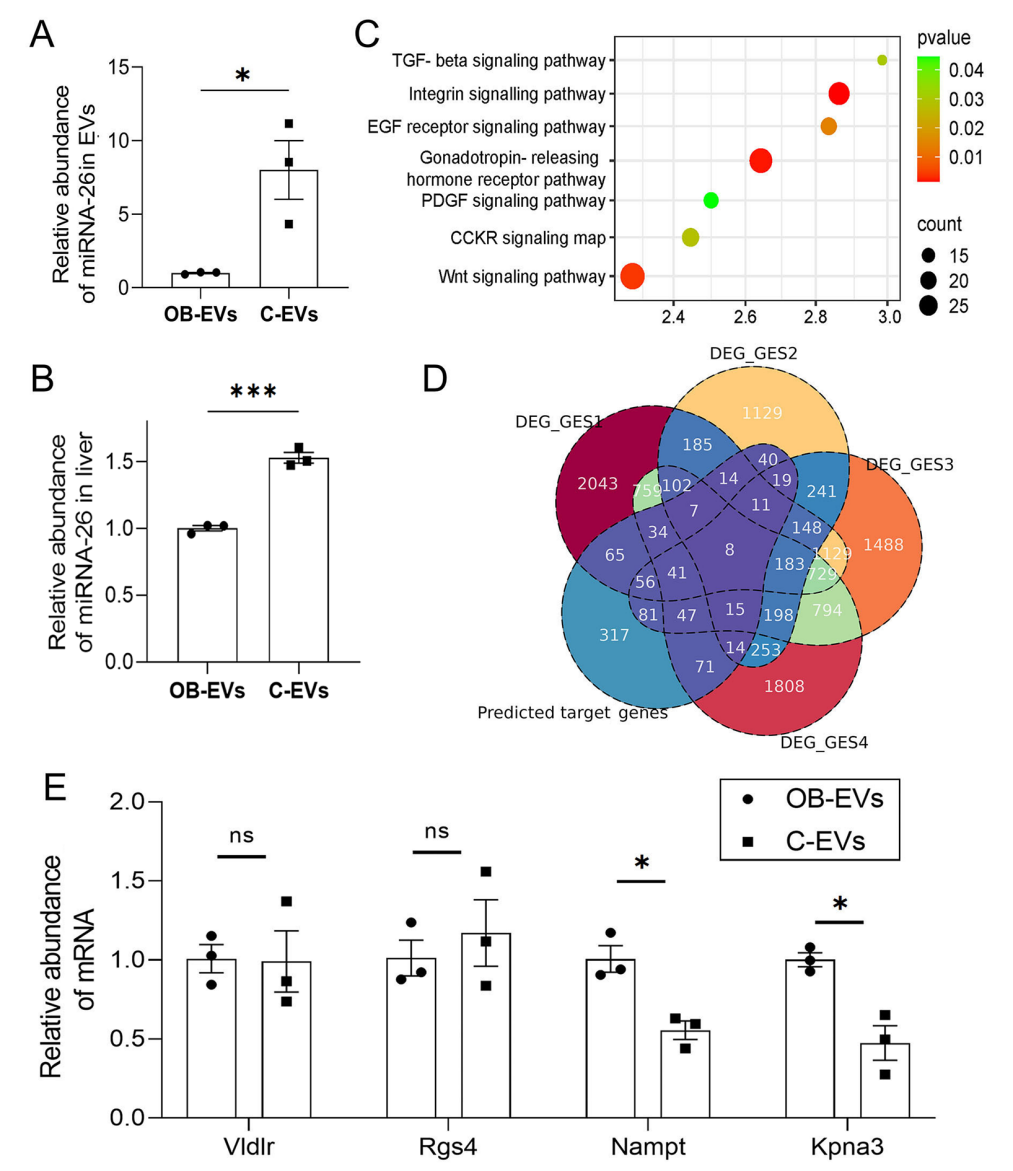
(A) Expression of miR-26 in plasma EVs from wild-type C57BL/6 and ob/ob mice. (B) Hepatic miR-26 levels in OB-EVs and C-EVs groups. (C) KEGG pathway enrichment bubble plot for predicted miR-26 target genes. Color gradient: *q*-value; circle size: number of target genes per pathway. (D) Venn diagram of overlapping genes between miR-26 targets and differentially expressed genes in ob/ob *vs.* wild-type livers (four independent datasets). (F) qPCR validation of hepatic VLDLR, RGS4, Nampt, and Kpna3 expression in OB-EVs and C-EVs groups.

Based on the discovery that administration of EVs from healthy mice improves hepatic lipid metabolism in obese mice, we sought to investigate the underlying molecular mechanisms. Given that the miR-26 family, has been mechanistically established as a key regulator of hepatic lipid metabolism and insulin sensitivity (Fu et al., 2015), we hypothesized miR-26a as a key mediator and prioritized its validation, and qPCR revealed that the expression level of miRNA-26a in C-EVs was significantly higher than that in EVs derived from ob/ob mice (Fig. 6A). Moreover, the hepatic miRNA-26a level in recipient ob/ob mice in the C-EVs group was significantly higher than that in the OB-EVs group, suggesting that EVs exert regulatory effects by delivering miRNA-26a to the liver (Fig. 6B). Predicted target genes of miRNA-26a were significantly enriched in pathways such as Wnt signaling (Fig. 6C). Further integration of four published liver transcriptome datasets (ob/ob *vs.* wild-type mice) identified seven predicted target genes of miRNA-26a that were differentially expressed in obese livers (Fig. 6D). qPCR validation of four predicted target genes (Vldlr, Rgs4, Nampt and Kpna3) of miRNA-26a showed that the mRNA expression levels of Nampt and Kpna3 in the livers of C-EVs recipients were significantly reduced, respectively, compared to the OB-EVs group (Figs. 6D–6E). These results suggest that EVs from healthy donors alleviate hepatic lipid accumulation and steatosis, potentially through delivering miRNA-26a, thereby targeting and suppressing the expression of Nampt (a rate-limiting enzyme in NAD+ synthesis, whose overexpression promotes hepatic lipid accumulation) and Kpna3 (involved in nuclear translocation of inflammatory signals and associated with the progression of steatohepatitis).

## Discussion

This study systematically evaluated the species-specific effects of EVs administration and their metabolic impacts, revealing significant differences in biocompatibility and immunogenicity between syngeneic and xenogeneic EVs. The findings demonstrate that homologous EVs (C57BL/6 to C57BL/6) induce only reversible physiological fluctuations under acute exposure (24 h), while long-term intervention (4 weeks) does not significantly alter blood biochemical parameters or hematopoietic system parameters, suggesting minimal acute toxicity in this syngeneic setting. In contrast, heterologous EVs (rabbit to mouse) triggered a significant increase in ALP and an imbalance in leukocyte subsets, suggesting that cross-species administration may activate innate immune responses. These phenomena can be attributed to the species-dependent molecular composition of EVs and their cargo. Through a cross-species EV administration model, this study revealed that xenogeneic EVs may induce innate immune activation, highlighting the need for cautious consideration of cross-species EVs in clinical applications and providing robust scientific evidence for the potential use of non-human derived EVs in human therapies.

Specifically, the conserved interaction between surface markers of homologous EVs (*e.g.*, CD47) and the SIRPα protein of the recipient species enables evasion of clearance by the mononuclear phagocyte system (Kamerkar et al., 2017; Kim et al., 2022). The CD47-SIRPα axis-mediated immune evasion strategy of EVs has been widely applied in tumor-targeted therapies (Hao et al., 2023; Kim et al., 2025). In contrast, heterologous transmembrane proteins (*e.g.*, MHC-I) carried by heterologous EVs may be recognized by pattern recognition receptors (*e.g.*, TLR4) (Xia et al., 2024), triggering neutrophil infiltration and ALP release, a process highly similar to the molecular mimicry mechanism in allogeneic administration (Fretwurst et al., 2018). Through a cross-species EV administration model, this study revealed that heterologous EVs may innate immune activation, highlighting the need for cautious consideration of cross-species EVs in clinical applications and providing robust scientific evidence for the use of porcine-derived EVs in human therapies (Hernandez et al., 2018).

Moreover, this study revealed that EVs derived from healthy donors significantly alleviated hepatic steatosis in ob/ob mice. The enrichment of miRNA-26a in these healthy donor EVs (C-EVs), and its subsequent increase in recipient livers, provides a plausible mechanistic link to this phenotypic improvement. This finding aligns with the established role of the miR-26 family as a key regulator of hepatic metabolism, wherein its overexpression has been shown to suppress lipogenic pathways and improve insulin sensitivity (Fu et al., 2015). In our study, this mechanism is further supported by the observed downregulation of Nampt, a predicted target of miRNA-26a. As a rate-limiting enzyme in NAD+ synthesis, Nampt has been implicated in promoting hepatic lipid accumulation, and its suppression represents a coherent, miR-26-mediated action that could directly contribute to the amelioration of steatosis. The therapeutic efficacy observed may be attributed to the involvement of miR-26 family members, particularly miR-26a, in mitigating metabolic dysregulation. This mechanism likely involves the modulation of lipid synthesis pathways, as supported by prior mechanistic studies (Fu et al., 2015; Guan, Chen & Fan, 2024; Chen et al., 2024). The beneficial effects are potentially mediated by the enrichment of miRNA-26a in C-EVs, which coincides with the downregulation of putative targets, including Nampt and Kpna3. However, direct mechanistic relationships warrant further validation.

This study has important implications spanning fundamental mechanisms to translational medicine. It systematically establishes the safety advantages of homologous EVs as therapeutic carriers for metabolic disorders, offering a solid foundation for prioritizing autologous or allogeneic EV-based therapies in clinical practice. EVs derived from healthy sources appear to be key regulators of metabolic enhancement, providing a basis for the development of multi-targeted metabolic intervention strategies based on EV technology. Additionally, the identification of potential immunogenicity risks associated with heterologous EVs suggests the need for careful re-evaluation of cross-species EV application standards within the international scientific community. These findings could help expedite the translation of EV-based therapies from conceptual validation to clinical implementation.

Our study has some limitations that, in turn, could serve as future avenues of investigation. Our EV preparations were isolated using ultracentrifugation, a robust but non-exclusive method that may co-isolate non-vesicular components. Although the observed biological effects are likely attributable to EVs, future studies employing techniques such as size-exclusion chromatography could further enhance preparation purity and confirm our findings. Due to experimental constraints, we did not perform biodistribution tracking of the administered EVs. Therefore, while we report systemic effects, the precise tissue tropism and cellular uptake efficiency of the EVs remain to be fully elucidated. The therapeutic mechanism of miR-26, while supported by correlation and target prediction, requires direct functional validation through gain- and loss-of-function experiments in the future. The experimental system, currently confined to rodent models, would benefit from expansion to non-human primates to validate the effects of prolonged EV exposure on immune and organ function. Furthermore, the targeting mechanism of miRNA-26a, primarily based on bioinformatics predictions and expression correlation analyses, needs functional validation through gene knockout or locked nucleic acid antagonism experiments. The current EV preparation method, relying on ultracentrifugation, may co-isolate non-EV vesicles such as apoptotic bodies, highlighting the potential need to integrate density gradient centrifugation and other techniques to enhance purity. Additionally, exploring the development of engineered EVs or combined drug delivery strategies could potentially improve metabolic regulation efficacy, representing an important area for further investigation.

## Conclusions

This study showed that syngeneic EV administration is safe and low-toxic, while xenogeneic EV administration may trigger immune responses. EVs from healthy donors effectively reduced hepatic steatosis in obese mice, supporting evidence for the clinical translation of EV-based therapies.

##  Supplemental Information

10.7717/peerj.20952/supp-1 Supplemental Information 1 Raw data

10.7717/peerj.20952/supp-2 Supplemental Information 2 MIQE Checklist

10.7717/peerj.20952/supp-3 Supplemental Information 3 Author Checklist

10.7717/peerj.20952/supp-4 Supplemental Information 4 Primer and Ct for qPCR

## Additional Information and Declarations

### Data Availability

The following information was supplied regarding data availability: The raw data are available in the Supplemental Files.
